# Neuroprotective effects of psilocybin in a rat model of stroke

**DOI:** 10.1186/s12868-024-00903-x

**Published:** 2024-10-08

**Authors:** Seong-Jin Yu, Kuo-Jen Wu, Yu-Syuan Wang, Eunkyung Bae, Fabio Chianelli, Nicholas Bambakidis, Yun Wang

**Affiliations:** 1 Center for Neuropsychiatric Research, National Health Research Institutes, Zhunan, Taiwan; 2 PharmaTher Inc, Toronto, ON Canada; 3 Department of Neurosurgery, University Hospitals of Cleveland, Cleveland, OH USA; 4 School of Pharmacy, College of Pharmacy, China Medical University, Taichung, Taiwan

**Keywords:** Stroke, Psilocybin, BDNF, Psychedelic

## Abstract

**Background:**

Psilocybin is a psychedelic 5HT2A receptor agonist found in “magic mushrooms”. Recent studies have indicated that 5HT2A agonists, such as dimethyltryptamine, given before middle cerebral artery occlusion (MCAo), improve staircase behavior, increased BDNF expression, and reduce brain infarction in stroke rats. The objective of this study is to determine the protective effect of psilocybin in cellular and animal models of stroke.

**Methods:**

Adult male and timed-pregnant Sprague-Dawley rats were used for this study. The neural protective effects of psilocybin were determined in primary rat cortical neurons and adult rats. Rats were subjected to a 60-min middle cerebral artery occlusion. Brain tissues were collected for histological and qRTPCR analysis.

**Results:**

Psilocybin reduced glutamate-mediated neuronal loss in rat primary cortical neuronal cultures. Psilocybin-mediated protection in culture was antagonized by the BDNF inhibitor ANA12. Pretreatment with psilocybin reduced brain infarction and neurological deficits in stroke rats. Early post-treatment with psilocybin improved locomotor behavior, upregulated the expression of MAP2 and synaptophysin, and down-regulated the expression of IBA1 in the stroke brain. ANA12 significantly attenuated psilocybin-mediated reduction in brain infarction and improvements in locomotor behavior.

**Conclusions:**

Psilocybin reduced brain infarction and improved locomotor behavior in stroke rats; the protective mechanisms involve regulating BDNF expression. Our data support a novel therapeutic approach of psilocybin in stroke.

## Highlights

Psilocybin reduced glutamate-mediated neuronal loss in primary cortical neuronal cultures.

ANA12 reduced psilocybin-mediated protection in culture.

Psilocybin reduced brain infarction, reduced neurological deficits, and increased locomotor behavior in stroke rats.

Psilocybin enhanced the expression of MAP2 and synaptophysin, and down-regulated IBA1 in the stroke rat brain.

## Background

Psilocybin (4-phosphoryloxy-N, N-dimethyltryptamine) is a psychoactive compound in wild or “divine” mushrooms *teonanacatl* initially used by Aztec Indians in Mexico for religious and healing rituals [1]. In the 16th century, Spanish Franciscan friar Bernardino de Sahagun first documented these mushrooms and referred *teronanacatl* as “God’s Flesh” or the sacred mushroom of Mesoamerica [2]. The active component Psilocybin was identified from the mushroom *Psilicybe Mexicana* and was named by Albert Hofmann [3]. Pharmacological effects of Psilocybin have been examined in animal studies since 1958 [4]. Both animal and clinical trials demonstrated its therapeutic effects for depression, anxiety, and addiction to alcohol or nicotine [5–8]. Sandoz has marketed the synthetic psilocybin (Indocybin) for experimental and psychotherapeutic purposes in the 1960s. Compass Pathways was granted a US Patent for treating drug-resistant depression with a psilocybin formulation in 2020.

Psilocybin is rapidly dephosphorylated to the active metabolite psilocin (4-hydroxy-N, N-dimethyltryptamine) after oral or intravenous administration [9, 10]. Psilocybin is water-soluble; psilocin is not water-soluble. Psilocin is thus more efficient than psilocybin in crossing the blood-brain barrier [11]. Both psilocin and psilocybin are serotonin (5-HT) receptor 2 A agonists. The psychedelic effect of psilocybin is antagonized by a 5-HT2A antagonist ketanserin, suggesting this response is mediated through the 5HT2A receptor [12]. Besides the 5HT2A pathway, other mechanisms are also involved in psilocybin-mediated responses. Recent studies have demonstrated that antidepressant-like behavioral and synaptic actions of psilocybin were independent of 5-HT2R activation in mice [13, 14].

Psilocybin is structurally similar to N, N-dimethyltryptamine (DMT, a 5HT2A psychedelic). Both psilocybin and DMT [15] increased synaptic density [16], improved neuroplasticity [17, 18], and showed anti-depressant [19] activity in experimental animals. The neuroplasticity action of DMT is associated with the expression of neurotrophins [18]. The TrkB (tyrosine receptor kinase B) inhibitor ANA12 or the mTOR inhibitor rapamycin antagonized DMT-mediated neuritogenesis or neuroplasticity in cortical neurons [15]. These data suggest that DMT improves synaptogenesis through BDNF/TrkB/ mTOR. Since psilocybin also increases the expression of BDNF protein in plasma [20], psilocybin may also induce trophic actions through BDNF.

Stroke is a major leading cause of death and adult disability. Tissue plasminogen activator (tPA) is the only US FDA -approved pharmacological therapy for acute ischemic stroke. tPA dissolves occlusive blood clots at an early stage of stroke. However, its effectiveness is limited by a narrow therapeutic time window. It is thus important to develop new therapies for stroke. Various pharmacological agents, including 5-HT2R agonists, have been examined for protection against stroke. In a study of a rat model of stroke, intraperitoneal injection of DMT before middle cerebral artery occlusion (MCAo) improved staircase behavior, increased BDNF expression, and reduced brain inflammation and infarction [21]. Although psilocybin and DMT are structurally alike, the protective effect of psilocybin in stroke has not been investigated.

Here we report that psilocybin reduced brain infarction and improved locomotor behavior in stroke rats. Our data support a novel therapeutic approach of psilocybin in stroke, which is often associated with subsequent depression.

## Materials and methods

### Animals and drugs

Adult male and timed-pregnant Sprague-Dawley rats were purchased from BioLASCO, Taipei, Taiwan. The use of animals was approved by the Animal Research Committee of the National Health Research Institutes of Taiwan (NHRI-IACUC- 109097-A). All experiment procedures, including housing and husbandry conditions, environmental enrichment, animal care/monitoring, and humane endpoints, were carried out in accordance with the National Institutes of Health Guidelines for the Care and Use of Laboratory Animals. As estrogen is protective against stroke [22–24] and hormonal changes during the menstrual cycle may interfere with behavioral and other physiological responses, the protective effect of psilocybin was examined in male animals in this study. ANA12 was purchased from Sigma-Aldrich (St. Louis, MO, USA), and psilocybin was purchased from Cayman Chemical (Ann Arbor, Michigan, USA). The use of this drug controlled substance psilocybin for the in vivo and in vitro studies was approved by the Taiwan FDA (approval number GRR09600000107).

### Primary cultures of rat cortical neurons (PCN)

Primary cultures were prepared as we previously described [25]. In brief, timed (E14–15) pregnant Sprague-Dawley rats were anesthetized and euthanized with a lethal dose of isoflurane. Embryonic cortex tissues were harvested and placed into cooled Dulbecco’s modified Eagle’s medium (Invitrogen). After rinsing off trypsin with pre-warmed Dulbecco’s modified Eagle’s medium, cells were dissociated by trituration, counted, and plated into 96-well (5.0 × 10^4^/well) cell culture plates precoated with Poly-D-Lysine (Sigma-Aldrich). The culture plating medium consisted of neurobasal medium supplemented with 2% heat-inactivated fetal bovine serum, 0.5 mM L-glutamine, 0.025 mM L-glutamate and 2% B27 (Invitrogen). Cultures were maintained at 37 °C in a humidified atmosphere (5% CO_2_ and 95% air). The cultures were fed by exchanging 50% of media with feed media (Neurobasal medium, Invitrogen) with 0.5 mM L-glutamate and 2% B27 with antioxidant supplements on days in vitro (DIV) 3 and 5. On DIV 7 and 10, cultures were fed with media containing B27 supplements without antioxidants (Invitrogen). Cultures were treated with reagents on DIV 10. After 48 h, cells were fixed with 4% paraformaldehyde (PFA) for 1 h at room temperature.

### Immunocytochemistry

After removing 4% PFA solution, cells were washed twice times with PBS and treated with a blocking buffer (5% BSA and 0.1% Triton X-100 in PBS) for 1 h. The fixed cells were incubated with a mouse monoclonal antibody against MAP2 (1:500; Millipore, Billerica, MA) at 4 °C for 1 day and were then rinsed three times in PBS. The bound primary antibody was visualized using AlexaFluor 488 goat anti-mouse secondary (Invitrogen). Images were acquired using a camera DS-Qi2 (Nikon, Melville, NY) attached to a NIKON ECLIPSE Ti2 (Nikon, Melville, NY). Data were analyzed by NIS-Elements AR 5.11 Software (Nikon). Controls consisted of omission of the primary antibody.

### Drug delivery and stroke surgery

Psilocybin was given before or after the stroke surgery. In the pretreatment study, psilocybin (100 µM x 20 µL, *n* = 7) or vehicle (saline, 20 µl, *n* = 7) was administered intracerebroventricularly (i.c.v., AP, − 0.8 mm; LV, − 1.5 mm; DV, − 3.5 mm) at 15 min before the right middle cerebral artery occlusion (MCAo). The speed of injection was controlled by a syringe pump (Micro 4, WPI, Sarasota, FL, USA). A subgroup of animals was used to examine the interaction of psilocybin and ANA12. These animals received psilocybin (100 µM x 20 µL, i.c.v.) 15 min before MCAo. Animals were anesthetized with isofluorane; ANA12 (100 µM x 20 µL/d, *n* = 7) or vehicle (20 µL/d, *n* = 7) was given intranasally after the MCAo on days 0 and 1 as previously described [26]. In the post-treatment group, psilocybin (1 mg/kg/day, *n* = 7) or vehicle (*n* = 6) was administered i.p. from day 3 to day 7. Animals were euthanized with a lethal dose of isoflurane and decapitated on day 9 for qRT-PCR analysis.

The right middle cerebral artery (MCA) was transiently occluded (MCAo) [27] with minor modifications as we previously described [28–30]. In brief, the bilateral common carotids were identified and clamped with nontraumatic arterial clips. The right MCA was ligated with a 10-O suture to generate focal infarction in the cerebral cortex. The clips and ligature were removed after 60 min ischemia to induce reperfusion injury. Core body temperature was maintained at 37 °C with a heating pad during anesthesia. After recovery from anesthesia, body temperature was maintained at 37 °C using a temperature-controlled incubator.

### Neurological tests

An elevated body asymmetry test was used to analyze body asymmetry [25, 31]. Rats were examined for lateral movements/turning when their bodies were suspended 20 cm above the testing table by lifting their tails. The frequency of initial turning of the head or upper body contralateral to the ischemic side was counted in 20 consecutive trials. In non-stroke rats, the average body asymmetry is 10 contralateral turns/20 trials (i.e., the animals turn in each direction with equal frequency). The maximum impairment in body asymmetry in stroke animals is 20 contralateral turns/20 trials.

Neurological deficits were further scored by the Bederson’s neurological test as previously described [32, 33]. In a postural reflex test, rats were examined for the degree of abnormal posture when suspended by 20–30 cm above the testing table. They were scored according to the following criteria.

0: Rats extend both forelimbs straight. No observable deficit.

1: Rats keep one forelimb to the breast and extend the other forelimb.

straight.

2: Rats show decreased resistance to lateral push in addition to behavior in score 1 without circling.

3: Rats twist the: upper half of their body in addition to behavior in score 2.

### Locomotor behavioral measurement

Locomotion was measured using an infrared activity monitor (Accuscan, Columbus, OH, USA) as we previously described [25, 34]. Rats were individually placed in a 3D infrared behavior chamber (42 × 42 × 21 cm) for 60 min. Four parameters were recorded: (a) horizontal activity (HACTV, the total number of beam interruptions that occurred in the horizontal sensors), (b) total distance traveled (TOTDIST, the distance, in centimeters, traveled by the animals), and (c) number of movements (MOVNO), (d) horizontal movements time (MOVTIME).

### 2,3,5-Triphenyl tetrazolium chloride (TTC) staining

The stroke animals were euthanized by lethal isoflurane and then decapitated. Brains were removed, immersed in cold saline for 5 min, and sliced into 2.0 mm thick sections. The brain sections were incubated in 2% TTC (Sigma-Aldrich) for 10 min and then transferred into a 5% formaldehyde solution for fixation. The area of infarction on each brain slice was measured double-blind using a digital scanner and the Image Tools program (University of Texas Health Sciences Center, San Antonio, TX, USA).

### Quantitative reverse transcription PCR (qRT-PCR)

The stroke animals were euthanized by lethal isoflurane and then decapitated. Cortical tissues were collected for qRT-PCR analysis. Total RNAs were isolated using TRIzol Reagent (ThermoFisher, Waltham, MA, USA); cDNAs were synthesized from 1 µg total RNA using RevertAid H Minus First Strand cDNA Synthesis Kit (ThermoFisher). IBA1, BDNF, MAP2, Synaptophysin (SYP), and GAPDH (ThermoFisher) were determined by specific universal probe library primer-probe sets or gene-specific primers (Table 1). Samples were mixed with TaqMan Fast Advanced Master Mix (Applied Biosystems, Waltham, MA USA) or SYBR (Luminaris Color HiGreen Low ROX qPCR Master Mix; ThermoScientific, ). qRT-PCR was carried out using the QuantStudio™ 3 Real-Time PCR System (ThermoScientific). The expression of the target genes was normalized relative to the reference gene (GAPDH) with a modified delta-delta-Ct algorithm. All experiments were carried out in duplicate.

**Table 1:** Oligonucleotide primers used for quantitative RT-PCR

Gene	SYBR Green		TagMan
	Forward	Reverse	
BDNF	CACTTTTGAGCACGTCATCG	TCCTTATGGTTTTCTTCGTTGG	
MAP2	CAAACGTCATTACTTTACAACTTGA	CAGCTGCCTCTGTGAGTGAG	
SYP	TTGGCTTCGTGAAGGTGCTGCA	ACTCTCCGTCTTGTTGGCACAC	
IBA1			Rn00574125_g1
GAPDH			Rn01775763_g1

### Randomization

Randomisation was used to allocate experimental units to control and treatment groups. The randomisation sequence was assigned through a computer.

### Statistical analysis

All data were included for statistical analysis. Data were presented as mean ± s.e.m. Student’s test, one or two–way ANOVA, and post-hoc Newman-Keuls test (NK test) were used for statistical comparisons. All analyses were calculated by Sigmaplot software ver 10.0. Statistical significance was defined as *p* < 0.05.

## Results

### Psilocybin dose-dependently reduced glutamate neurotoxicity in primary cortical neuronal culture

**Fig. 1:**
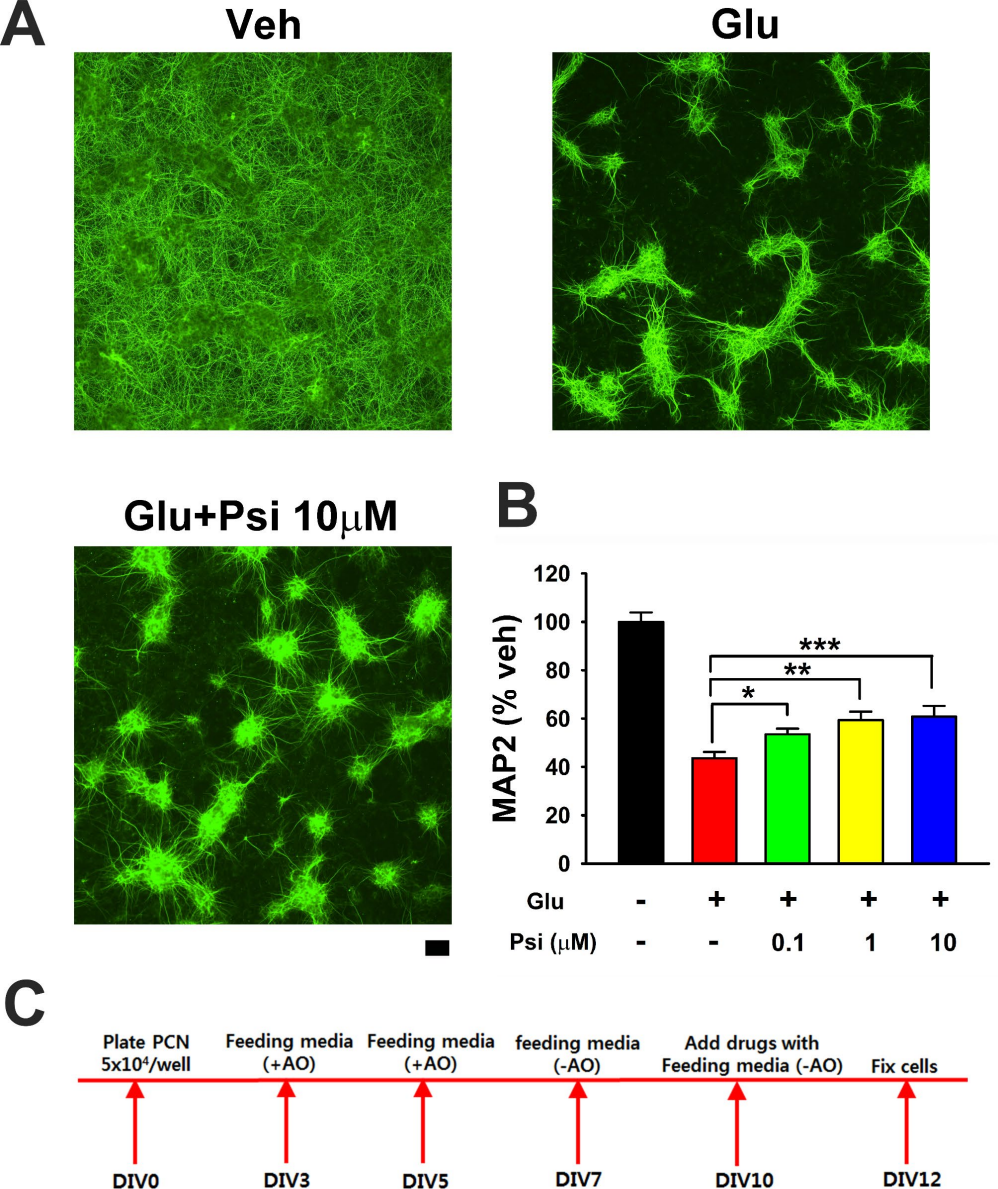
Psilocybin (Psi) dose-dependently reduced glutamate-mediated neuronal loss in rat primary cortical neuronal cultures. (**A**) Representing photomicrographs indicate that glutamate (Glu, 100µM) reduced MAP2 immunoreactivity (MAP2-ir). Glu-mediated neuronal loss was antagonized by psilocybin (Psi, 10µM). (**B**) MAP2-ir from all studies was averaged and analyzed. Glu significantly reduced MAP2-ir (p<0.001, F_4,35_=42.103, one-way ANOVA+NK test). Psi dose-dependently mitigated this degenerative response (*p=0.010, **p=0.011, **p=0.003, number of wells=6–12 per group, F_4,35_=42.103, one-way ANOVA+NK test). (**C**) Timeline. +AO: with antioxidant. -AO: without antioxidant. Calibration: 100 µm

We first examined the protective effect of psilocybin in primary neuronal cultures. Glutamate, psilocybin, and control vehicle were added to the primary cortical culture on DIV10 (day-in-vitro, see timeline in Fig. 1C). A high dose of glutamate (100 µM) was used to produce neurodegeneration and simulate elevated glutamate overflow during cerebral ischemia [35, 36]. Cells were fixed for Microtubule-Associated Protein 2 immunocytochemistry (MAP2) on DIV12. Glutamate significantly reduced MAP2 immunoreactivity (MAP-ir, Fig. 1A and B, *p* < 0.001). Psilocybin dose-dependently mitigated this reduction (Fig. 1B, *p* < 0.05, one-way ANOVA).

### ANA12 reduced psilocybin-mediated neuroprotection in vitro

**Fig. 2:**
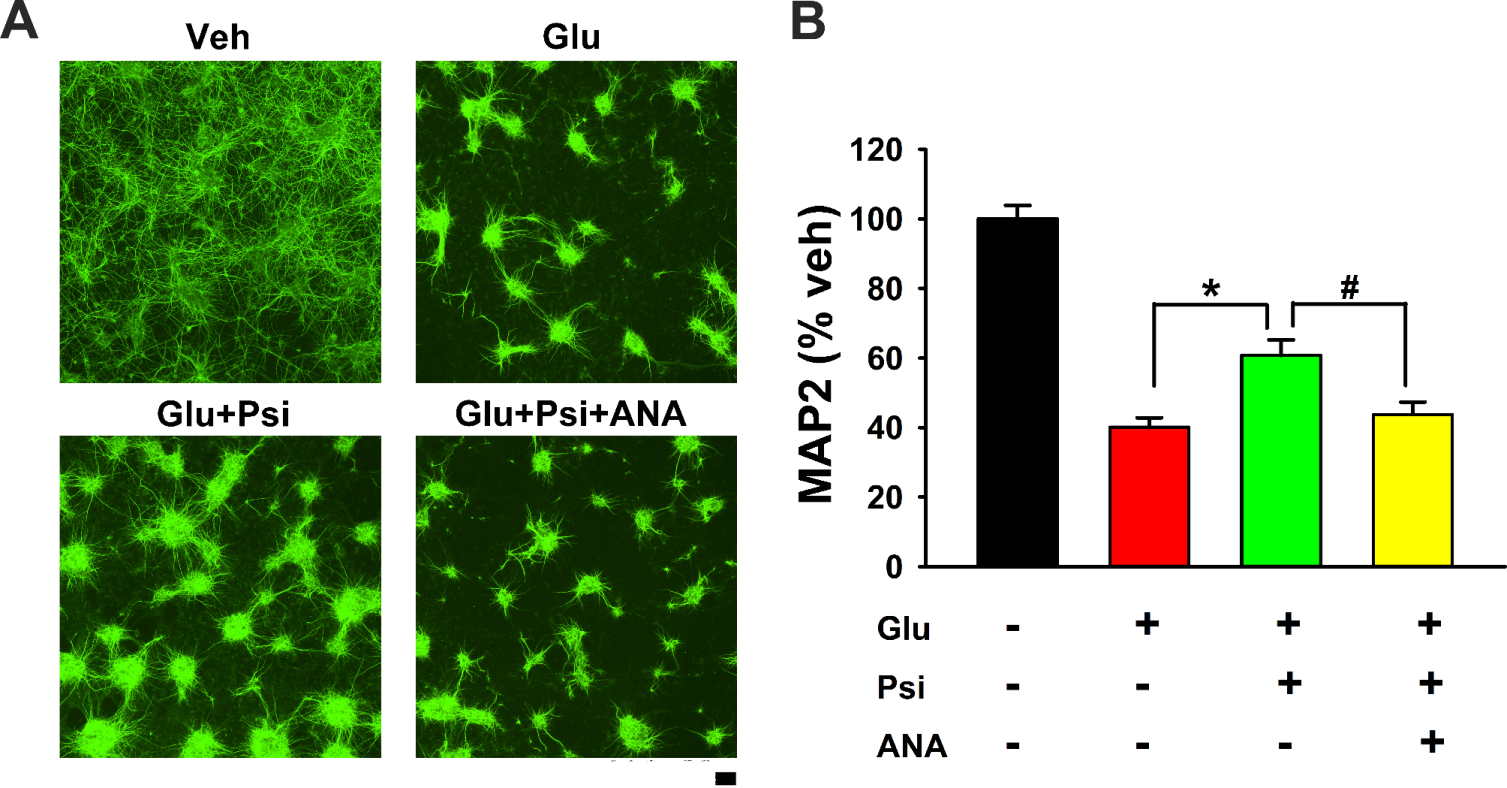
ANA12 antagonized psilocybin-mediated protection in primary neuronal culture. (**A**) Representing MAP2 immunostaining demonstrates that psilocybin (Psi, 10µM) antagonized Glu (100µM) -mediated neuronal loss. The improvement in MAP2 -ir was mitigated by ANA12 (ANA, 10 µM). (**B**) MAP2 -ir was analyzed in all wells (n=6 per group). Psi antagonized Glu -mediated neuronal loss (*p=0.002, F_3,20_=53.940). ANA12 significantly antagonized Psi -mediated protection (Glu+Psi vs. Glu+Psi+ANA, #p=0.004, one-way ANOVA+NK test). Calibration: 100 µm

We next examined if the protective response of psilocybin involved BDNF in primary neuronal culture. A selective TrkB antagonist ANA12 [20] or vehicle was co-administered with psilocybin and glutamate on DIV10 (see timeline in Fig. 1C). ANA12 significantly attenuated psilocybin-mediated improvement in MAP2 -ir (Fig. 2A and B, *p* = 0.004, one-way ANOVA + NK test).

### Intracerebral administration of psilocybin reduced behavioral deficits in stroke rats

**Fig. 3:**
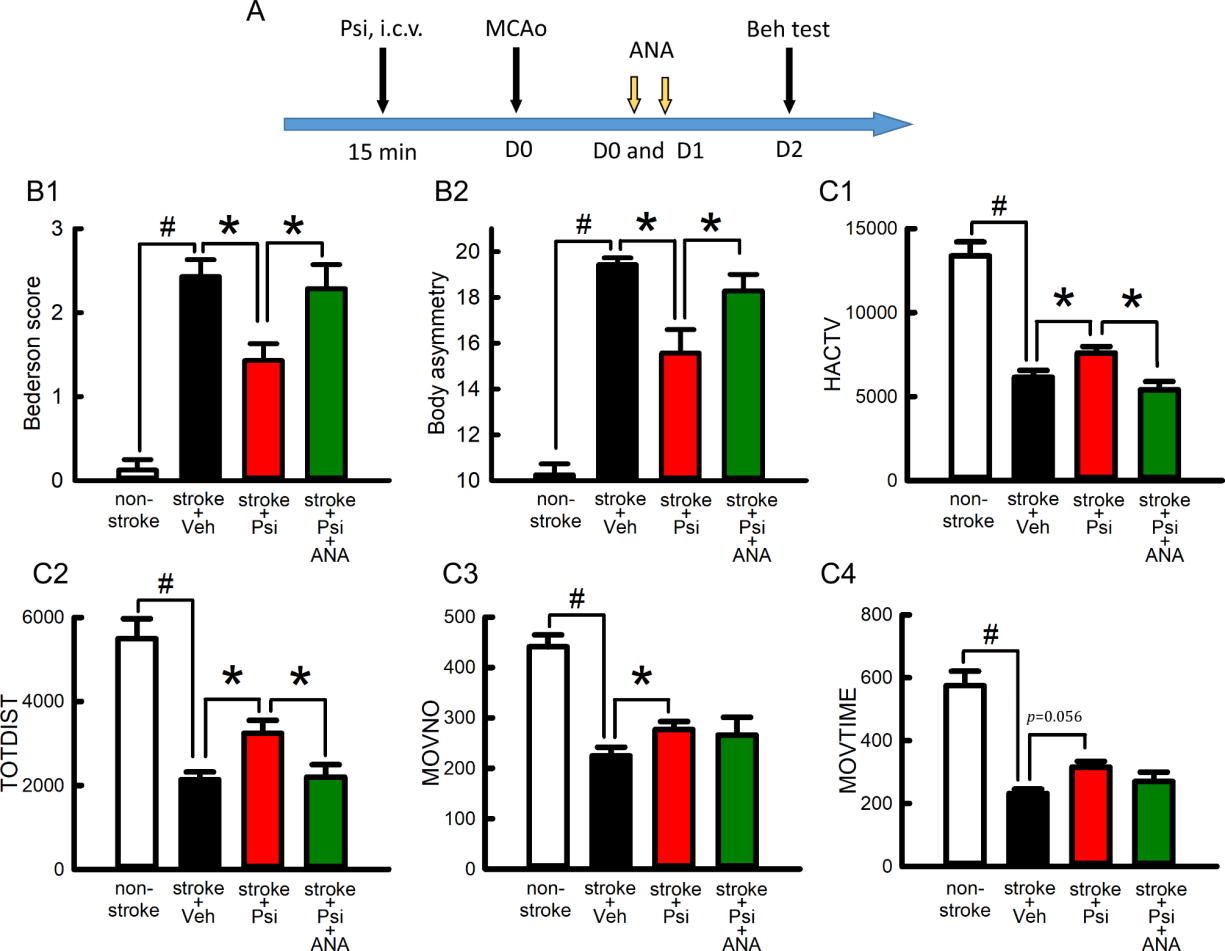
Pretreatment with psilocybin reduced behavioral deficits in stroke rats. ANA12 antagonized psilocybin-mediated behavioral improvement. (**A**) Timeline. Psilocybin (Psi) was given (i.c.v.) 15 min before the MCAo on D0. ANA12 was delivered intranasally after the MCAo on days 0 and 1. Behavioral tests were conducted on D2. Cerebal ischemia significantly (#p<0.001) increased (B1) Bederson’s neurological score (F_3,21_=16.411) and (B2) body asymmetry (F_3,21_=23.904), while reduced locomotor activity (C1, HACTV, F_3,21_=39.745; C2, TOTDIST, F_3,21_=20.506; C3, MOVNO, F_3,21_=23.904; C4, MOVETIME, F_3,21_=10.713). These behavior responses were significantly antagonized (*p<0.05) by Psi (B1, Bederson’s score, F_3,21_=16.411; B2, body asymmetry, F_3,21_=23.904; C1, HACTV, F_3,21_=39.745; C2,TOTDIST, F_3,21_=20.506; C3, MOVNO, F_3,21_=23.904; C4, MOVETIME, F_3,21_=10.713). ANA12 significantly (*p<0.05) antagonized Psi-mediated behavioral improvement (B1, B2, C1, C2). [one-way ANOVA+NK test; HACTV: horizontal activity; TOTDIST: total distance traveled; MOVNO: movement number; MOVETIME: movement time]

As psilocybin was neuroprotective in primary neuronal culture, we next examined whether a similar protective response was found in vivo. A total of 18 adult rats were used for this analysis. Of these, 14 rats received a 60-min MCAo, and 4 were used as non-stroke control. Psilocybin (100 µM x 20 µL, number of animals = 7) or vehicle (saline, 20 µL, *n* = 7) was delivered into the lateral ventricle (i.c.v.) 15 min before stroke surgery. Two behavioral tests were conducted two days (or D2) after the MCAo (Fig. 3A). (A) Neurological deficits were evaluated using Bederson’s neurological test and body asymmetry. Similar to previous reports, stroke animals had a significantly increased Bederson’s score and body asymmetry (Fig. 3B1, B2, *p* < 0.001, non-stroke vs. stroke). Psilocybin significantly reduced the Bederson’s neurological score (Fig. 3-B1, *p* = 0.011, one-Way ANOVA + NK test) and body asymmetry (Fig. 3-B2, *p* = 0.002, stroke + veh vs. stroke + Psi). (B) Locomotor activity was monitored for one hour on day 2. Locomotor activity was significantly reduced in stroke animals (Fig. 3-C1-4; *p* < 0.001, stroke vs. non-stroke). Psilocybin increased horizontal activity (HACTV, *p* = 0.039), total distance traveled (TOTODIST, *p* = 0.032), and movement number (MOVNO, *p* = 0.0284) as well as marginally increased movement time (MOVTIME, *p* = 0.056; Fig. 3C1-4). These data suggest that psilocybin, given intracerebroventricularly, reduces neurological deficits and increases locomotor activity in stroke rats.

**Fig. 4:**
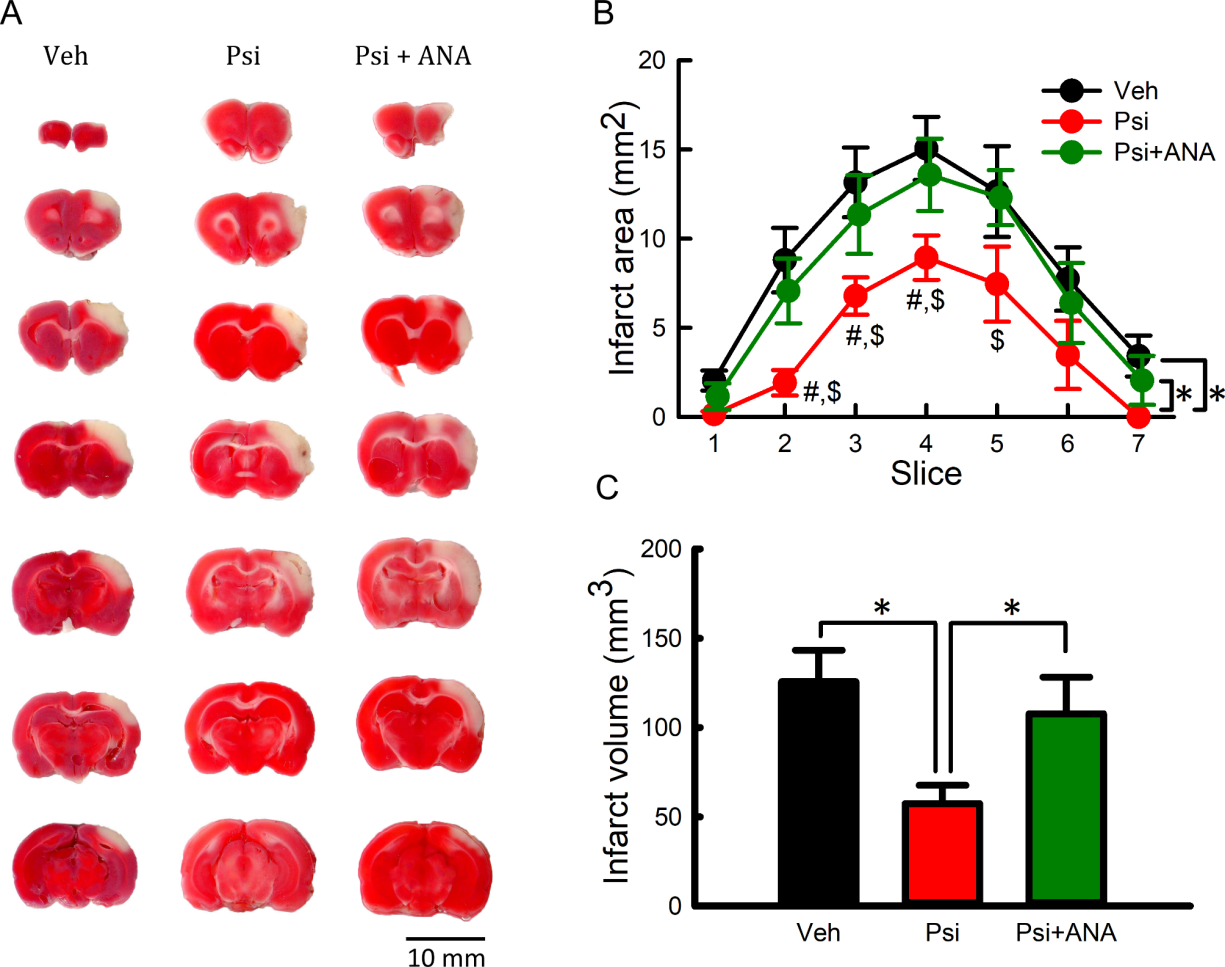
Pretreatment with psilocybin reduced brain infarction in stroke rats. ANA-12 significantly antagonized psilocybin-mediated protection. (**A**) Representing TTC images. (**B**) The area of infarction per 2-mm slices from rostral (slice 1) to caudal (slice 7) was quantified for all animals (n=7 per group). Psi significantly reduced infarction (*p<0.001, F=17.083, 2-way ANOVA). ANA antagonized Psi -mediated protection (*p<0.001). Posthoc NK test indicates that these changes mainly occurred between slice 2 and slice 5 (#p<0.05, Psi vs. veh; $p<0.05, Psi vs. Psi+ANA). (**C**) Psi significantly reduced infarct volume (*p=0.025, F_2,18_=4.507, one-Way ANOVA+NK test). ANA12 antagonized Psi-mediated protection (*p=0.047)

### ANA12 attenuated psilocybin-mediated behavioral recovery in vivo

We next examined if the in vivo protective response of psilocybin involved BDNF. ANA12 was delivered intranasally after psilocybin injection on days 0 and 1 (100 µM x 20 µL/d x 2 d, *n* = 7, see Timeline, Fig. 3A) in stroke rats. ANA12 significantly reduced the psilocybin-mediated improvement in neurological behavior (Fig. 3, B1: *p* = 0.012; B2: *p* = 0.012), horizontal activity (Fig. 3, C1, *p* = 0.009, one-way ANOVA + NK test), and total distance traveled (Fig. 3C2, *p* = 0.018). These data suggest that psilocybin-improved behavioral function is mediated through BDNF.

### Intracerebroventricular administration of psilocybin reduced brain infarction

Brain tissues from stroke rats were harvested, sliced into 2 mm sections, and stained with TTC for infarction analysis on D2. As seen in the representative histological images (Fig. 4A, Psi vs. veh), psilocybin (i.c.v.) reduced brain infarction and ANA12 antagonized this psilocybin-induced protection. We next analyzed the distribution of the infarct area from rostral to caudal (Fig. 4B, #1 rostral to #7 caudal) in all animals. Psilocybin significantly reduced infarction area, mainly in the rostral brain (Fig. 4B, *p* < 0.001, two-way ANOVA + NK test). Similarly, psilocybin significantly reduced the volume of infarction (sum of infarct area per slice x thickness of slice; Fig. 4C, *p* = 0.025, one-way ANOVA + NK test). These protective responses of psilocybin in vivo were antagonized by ANA12 (Fig. 4B, *p* < 0.001; Fig. 4C, *p* = 0.047).

**Fig. 5:**
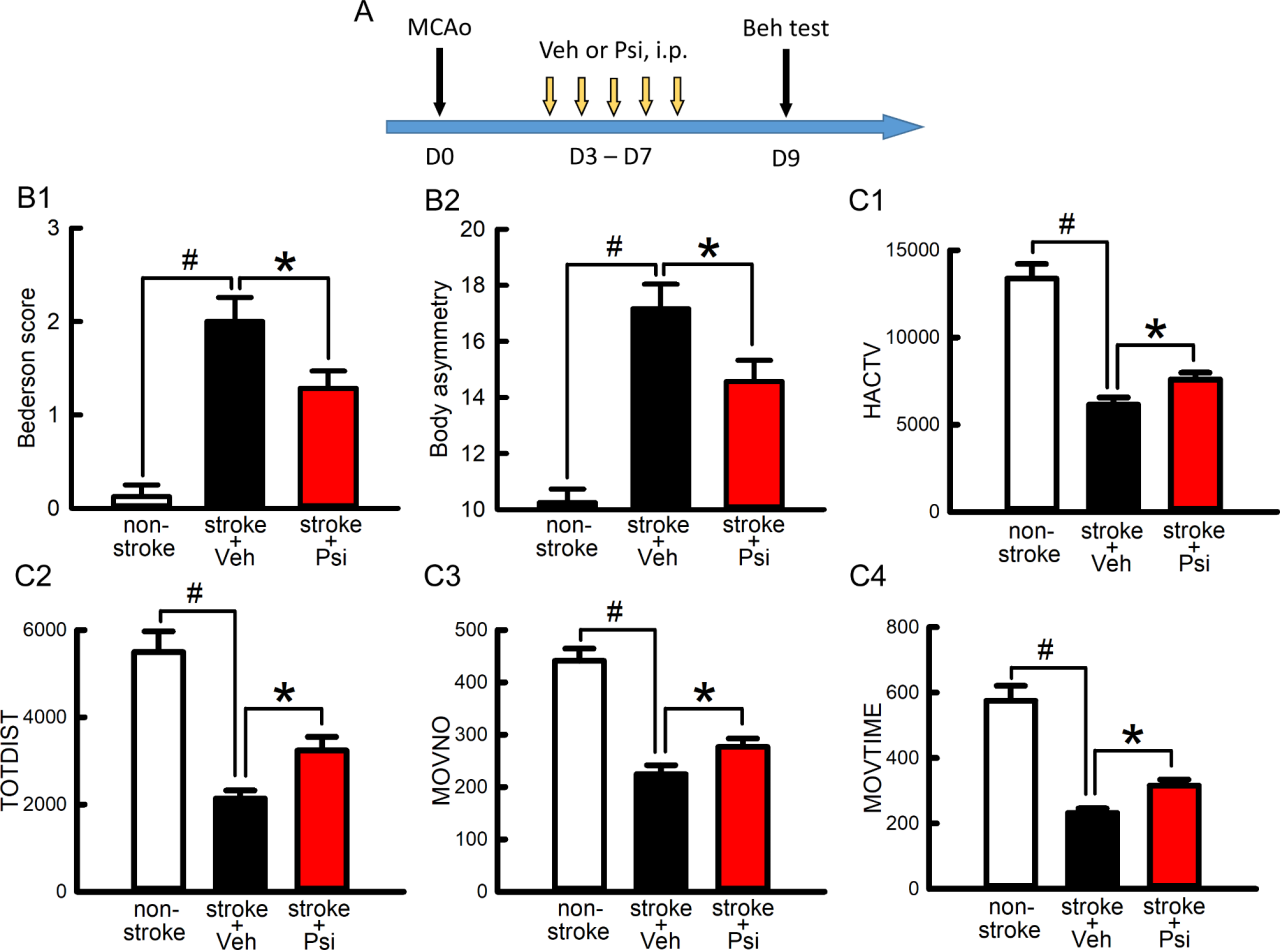
Early post-treatment with psilocybin improved behavior function in stroke rats. (**A**) Timeline. Psilocybin (Psi, 1mg/kg/d, n=7) or vehicle (n=6) was given (i.p.) daily between days 3 and 7 after the MCAo. Neurological and locomotor tests were conducted on day 9. Psi post-treatment significantly reduced (B1) Bederson’s neurological score (F_2,14_=16.344) and (B2) body asymmetry (F_2,14_=16.321) while increasing locomotor behavior [C1-4: HACTV (F_2,14_=6.887), TOTDIST (F_2,14_=5.848), MOVNO (F_2,14_=4.883), MOVTIME (F_2,14_=7.691)]. #p<0.001, *p<0.05, one-Way ANOVA+NK test

**Fig. 6:**
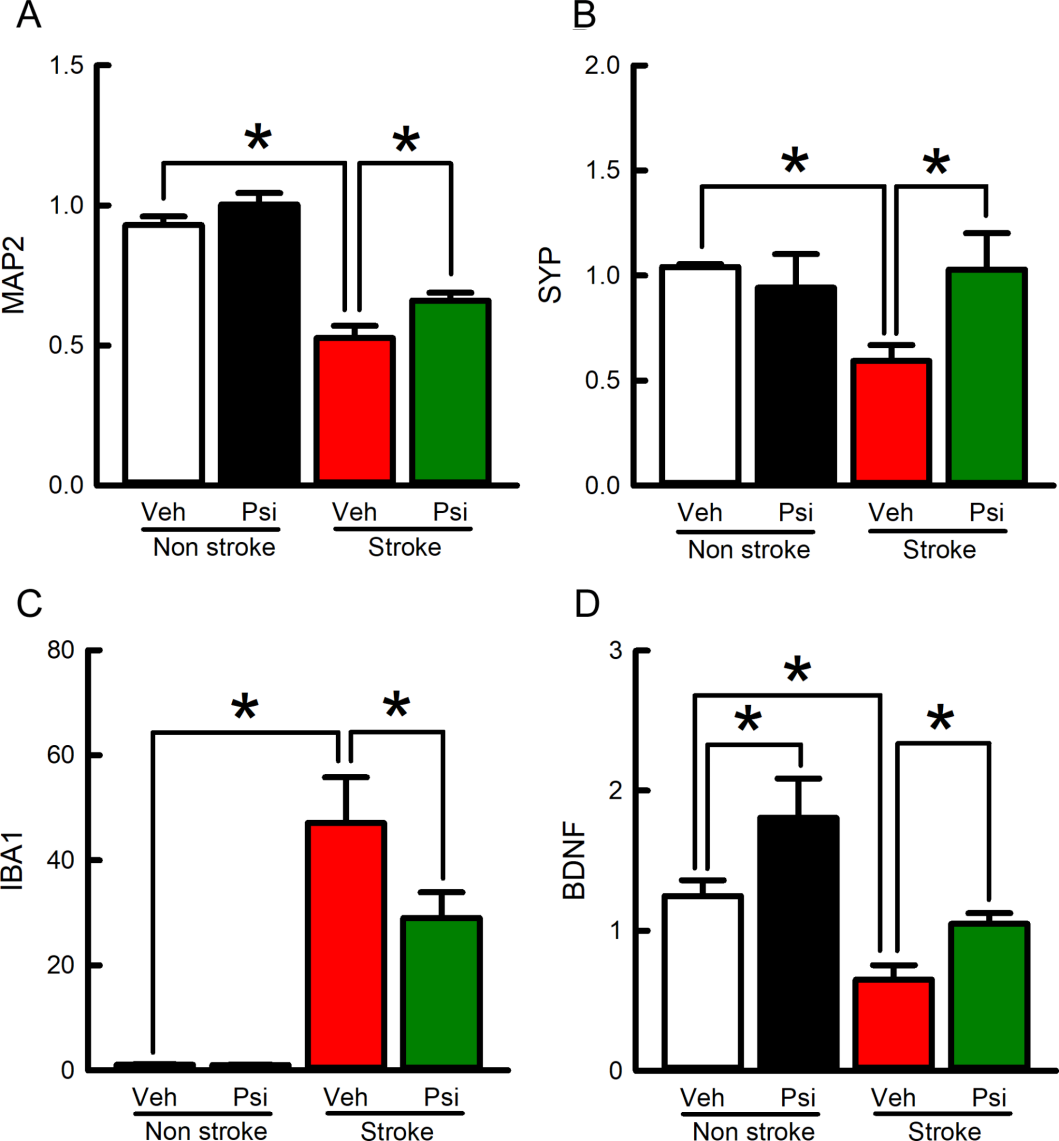
Post-stroke treatment with psilocybin upregulated the expression of neuronal markers and BDNF. Stroke and non-stroke side cerebral cortices were collected for qRTPCR analysis. (**A**-**C**) In the stroke side cortices, MAP2 and synaptophysin (Syn) were significantly downregulated while IBA1 was upregulated (MAP2: p<0.001, F=104.947; Syn: p=0.016, q=3.706, IBA1: p<0.001, F=59.635, stroke vs. non-stroke); these responses were antagonized by psilocybin (Psi+stroke vs. veh+stroke; MAP2: p=0.017; Syn: p=0.014; IBA1: p=0.014). (**D**) The expression of BDNF in the stroke (p=0.048) and non-stroke (p=0.033) brains was upregulated by Psi (F=9.558). Two-way ANOVA+NK-test

### Post-stroke treatment with psilocybin reduced neurological deficits and improved locomotor behavior

To examine if psilocybin has a similar protective effect given after stroke, psilocybin (1 mg/kg/d, *n* = 7) or vehicle (*n* = 6) was delivered systemically (i.p.) from days 3 to 7 after the MCAo. Four non-stroke rats were used as naive controls. Behavioral tests were conducted on day 9 (see timeline, Fig. 5A). Psilocybin significantly reduced neurological deficits (Fig. 5B1, Bederson’s neurological score, *p* = 0.024; B2: body asymmetry, *p* = 0.026, one-way ANOVA + NK test) while it increased locomotor behavior (Fig. 5-C1: horizontal activity *p* = 0.009; C2: total distance traveled, *p* = 0.009; C3: movement number, *p* = 0.022; C4: movement time, *p* = 0.02). These data suggest that early post-treatment with psilocybin improves behavioral function in stroke rats.

### Psilocybin reduced stroke-mediated changes in MAP2, synaptophysin, and IBA1

Stroke rats receiving post-treatment with psilocybin (*n* = 7) or vehicle (*n* = 6) were sacrificed on day 9. Bilateral cortical tissues were collected for neuronal (MAP2 and synaptophysin) and inflammatory marker (IBA1) analysis by qRTPCR. In the stroke side cortices, MAP2 (Fig. 6A, *p* < 0.001) and synaptophysin (Fig. 6B, *p* = 0.016) were significantly downregulated while IBA1 was upregulated (Fig. 6C, *p* < 0.001; stroke vs. non-stroke, two-way ANOVA + NK test). These marker responses to stroke were antagonized by psilocybin (Fig. 6A-C : Psi + stroke vs. veh + stroke; MAP2: *p* = 0.017; Syn: *p* = 0.014; IBA1: *p* = 0.014, two-way ANOVA + NK-test). Psilocybin significantly upregulated the expression of BDNF in stroke (Fig. 6D, *p* = 0.048) and non-stroke side cortices (*p* = 0.033, two-way ANOVA + NK-test).

## Discussion

We characterized the protective effects of psilocybin in neuronal cultures and in an animal model of stroke. Psilocybin reduced the glutamate-mediated loss of MAP2-ir in primary neuronal cultures. Parallel neuroprotective effects were seen in experimental animals. We demonstrated that pre- or post-treatment with psilocybin significantly reduced neurological deficits, increased locomotor behavioral function, reduced brain infarction, and suppressed expression of inflammatory genes in stroke rats. The main finding of this study is that psilocybin is a potent neuroprotective agent against ischemic stroke in animals. To clarify the protective effect of psilocybin in the CNS, we first administered the drug intracerebroventricularly in a pre-stroke study. We found that pre-stroke ICV psilocybin protected against ischemic brain injury. In the post-stroke experiment, psilocybin was given intraperitoneally from days 3 to 7. In both pre- and post- stroke studies, psilocybin significantly reduced neurological deficits. Bederson’s neurological score and body asymmetry were significantly improved. Animals showed a significant increase in locomotor activity. These data suggest that psilocybin, given pre- or early post- MCAo, improved motor behaviors in stroke rats. Previous studies have indicated a correlation between the infarction volume and body asymmetry or locomotor deficits in stroke rodents [34]. Together with these behavioral changes, psilocybin reduced brain infarction and increased the expression of neuronal markers (i.e., MAP2 and synaptophysin) in the lesioned brain, suggesting that psilocybin attenuates neurodegenerative changes after stroke.

One report in the literature indicated that the elevated body asymmetry test (EBST) is inconsistent in rats receiving proximal MCAo [37]. In contrast, our previous study demonstrated a significant correlation between the infarction volume and body asymmetry in animals receiving distal MCAo [34]. These differences may be attributed to the method of brain ischemia and outcomes (i.e., region of lesion, mortality) between these two models. For example, in the proximal MCAo model, the unilateral common carotid artery (CCA) and external carotid artery (ECA) were permanently ligated with a suture. Another nylon suture was inserted from the common carotid artery and advanced up the internal carotid artery. As ECA supplies facial tissues, ligation can cause ischemia in this region and confound the results of EBST. In our study, we used distal MCAo. The right middle cerebral artery was occluded for 60 min by ligation at its distal branch with a 10-O suture. ECA and CCA were not unilaterally and permanently occluded. Facial blood supply was thus less affected by the distal MCAo. Proximal MCAo generated infarction in the striatum and cortex by filament insertion, while distal MCAo in our study generated infarction mainly in the cortex (see Fig. 4A). The different brain regions of infarction may contribute to the behavioral difference between these two models. There is a high mortality after proximal MCAo. For example, 60 min occlusion generated 50% mortality in male rats [37]. In our study, we have a low mortality rate (close to 0) after 60 min distal MCAo.

Psilocybin is dephosphorylated to the active metabolite psilocin by systemic alkaline phosphatase after peripheral administration [11, 38]. Tissue non-specific alkaline phosphatase (TNAP) is also expressed in brain endothelial cells and involves the integrity of blood-brain barrier [39]. Psilocin may thus be present in brain and involve neuroprotection against ischemic brain injury after i.c.v. or i.p. administration of psilocybin.

After the onset of stroke, a series of progressive neurodegenerative steps are activated, leading to cell death, and affecting neural repair. Neuroinflammation is a major degenerative reaction. Ischemic insults activate innate microglia, enhance the production of cytokines and chemokines, and increase the infiltration of peripheral immune cells [40]. We and others previously reported that suppressing inflammation with pharmacological agents reduces neurodegeneration and improves behavioral recovery from ischemic injury [25, 41, 42]. In this study, post-treatment with psilocybin suppressed the expression of IBA1 and improved motor function. The anti-inflammatory effect of psilocybin in our study is further supported by recent reports that the extracts of psilocybin-containing mushrooms downregulated the expression of pro-inflammatory mediators [43, 44].

Previous clinical reports have suggested that psilocybin increased plasma BDNF levels in healthy subjects [20, 45]. BDNF regulates neuroprotection, neuroplasticity, and repair after ischemic brain injury [46, 47]. We found that psilocybin increased the expression of BDNF in the stroke brain. To further identify the role of BDNF in psilocybin-mediated protection, a TrkB inhibitor ANA12 was used [48] in both the in vitro and vivo experiments. ANA12 significantly antagonized psilocybin-mediated protection in MAP2-ir in primary neuronal culture and psilocybin-mediated improvements in brain infarction and locomotor behavior. These data suggest that BDNF is involved in the protective action of psilocybin in stroke brain.

The dose of psilocybin (1 mg/kg, i.p.) used in the systemic study was based on previous reports [49, 50]. Psilocybin, at 1 mg/kg, had no sedative effects but significantly reduced alcohol relapse in rats [50]. The doses (> or = 1 mg/kg) for rats used in our and other laboratories [50, 51] were higher than those reported in human studies [52]. For example, 18 to 59 mg (or 0.3–0.6 mg/kg/d) of psilocybin produced well-being or life satisfaction in healthy human participants [52]. The need for higher doses in stroke rats is not known and warrant further investigation. It has been reported that the activities of hepatic and intestinal isoforms of alkaline phosphatases in rat serum differ significantly from humans [53]. The differential pharmacokinetic or dynamic properties in rats, the disease models, and the endpoints of study may contribute to the variability in doses. Future studies are needed to clarify the therapeutic effect of psilocybin for stroke at lower doses or other dosing strategies.

tPA is currently the only FDA-approved therapy for ischemic stroke. tPA dissolves blood clots in the cerebral vessels. However, tPA has to be given within 3–4.5 h after the onset of stroke. Less than 3% of stroke patients receive tPA because they do not arrive at a hospital early enough for treatment. Our data suggest that pretreatment with psilocybin reduced brain infarction and neurological deficits. The effectiveness of pretreatment in stroke may be clinically useful for patients susceptible to ischemic events, for example, those suffering from transient ischemic attacks. We further demonstrated that post-treatment with psilocybin from days 3 to 7 after the MCAo reduced brain inflammation, upregulated neuronal markers and improved locomotor behaviors in stroke rats. It is thus likely that psilocybin has a relatively wider therapeutic time window.

In conclusion, we demonstrated that psilocybin is neuroprotective against ischemic brain injury—early post-treatment with psilocybin reduced neurodegeneration, inflammation, and neurological deficits in stroke animals. The mechanism of protection involved anti-inflammation and upregulation of the protective neurotrophic factor BDNF. Recent clinical studies have supported the antidepressant and anxiolytic effects of psilocybin in patients [54–56]. Psilocybin is currently under clinical trials for depression and anxiety (see clinicaltrials.gov). In 2019 the CDC tagged psilocybin as a “Breakthrough therapy for treatment-resistant depression.” As stroke patients have a higher incidence of depression and anxiety [57, 58], psilocybin may be a beneficial therapeutic agent to prevent or treat comorbidity of stroke and depression in patients.

## Data Availability

No datasets were generated or analysed during the current study.

## Abbreviations

BDNF: Brain-derived neurotrophic factor
IBA1: Ionized calcium binding adaptor molecule 1
i.c.v.: intracerebroventricularly
MAP2: Microtubule-associated protein 2
MCAo: middle cerebral artery occlusion
PCN: Primary cultures of rat cortical neurons
qRT-PCR: Quantitative reverse transcription PCR
SYP: Synaptophysin
TrkB: tyrosine receptor kinase B
TTC: 2,3,5-Triphenyl tetrazolium chloride
